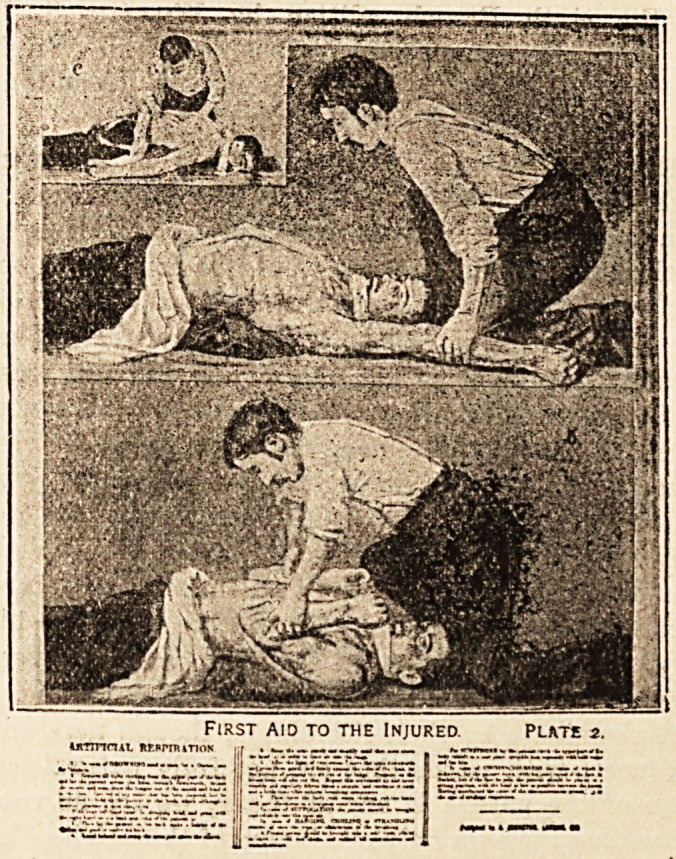# "The Hospital" Medical Book Supplement No. XXXIV

**Published:** 1910-10-01

**Authors:** 


### "THE HOSPITAL" MEDICAL BOOK SUPPLEMENT No. XXXIV.

### THE PRACTITIONER'S LIBRARY.

### SOME USEFUL NEW BOOKS AND WHERE TO GET THEM.

The formation of a practitioner's library is not always an easy task, especially to those who do not possess first-hand information about the various books suitable for such a collection. A mere glance at a publisher's catalogue is not sufficient. Naturally the wares are presented therein in the most attractive fashion. A work which may not be of the slightest use to the practising physician, an abstruse and involved monograph on a rare subject, or a volume dealing with commonplace lesions in so uninteresting and unattractive a manner that it repels the ordinary reader with any sense of literary decency, is often represented by illustrations and excerpts from reviews as being the best of its kind. The present short article is an attempt to help the practitioner in selecting works which will be of real practical service. None of the books here recommended are unworthy of attention. They are, above all, eminently practical, and their choice has been made after a careful comparison with many rivals in the field, solely with a view to the requirements of the general practitioner. The ideal book for this class of reader must fulfil many requirements. It must be handy, compact, and strongly bound, well printed, up to date, and pre-eminently a reference or "reminder" book. While it is desirable that it should give details of the newest and best methods, it should at the same time never forget that the practitioner is above all desirous of hearing what are the advantages of methods with which he himself is—or was in his hospital days—well acquainted, and what are the comparative merits of various methods. For that reason we think it is a mistake to buy American books, however well printed and published they are. There are many excellent manuals by American authors, but so far as we are aware they present no advantages over corresponding books by English authors or over good translations by Continental writers. Those intended for general practitioners are usually—and we speak from a comparison of a large number of works—far too elementary to be appreciated by the average English doctor, while the monographs, on the other hand, can scarcely compare, in fulness and admirableness, with similar productions emanating from Continental houses. At the same time, it must be remembered that there are some good translations of French and German books published by American houses. While these cannot honestly be recommended to practitioners who are able to read the originals, they are yet worthy the attention of those who wish a fair English version of a well-known standard work. Among these we may here mention Sahlis' "Diagnosis" and Nothnagel's monumental "Practice of Medicine," both very excellent works, which are suitable for the practitioner's library, though again we would advise "where you can obtain and read the original work do not be content with a translation, however excellent."

#### The Oxford Medical Manuals.

Of recent years the Oxford Medical Publications, for which a combined firm, Messrs. Hodder & Stoughton and Henry Frowde, is responsible, have been attracting deserved attention from practitioners. The medical editor, Mr. Keogh Murphy, has been at pains to select thoroughly practical works for inclusion in the series. One of the latest issues is Mr. Pringle's excellent book on the "Modern Treatment of Fractures." The following works issued by this firm may be instanced as throroughly suitable for the practitioner's library: "A System of Operative Surgery by British Surgeons," edited by F. F. Burghard, undoubtedly the premier work on operative surgery published in England, in four volumes at £6 the set complete; Osler and McCrae's "System of Medicine," a "practice" which vies with Allbutt in popularity; Wrench and Tweedy's "Practical Obstetrics," 12s. 6d. net; Wallace's "Prostatic Enlargement"; Sargent and Russell's "Emergencies of General Practice," and the sister volume, Corner and Pinches' "Operations of General Practice," new editions of which, both priced at 15s. net, have been issued recently, Corner's "Male Diseases in General Practice," at the same price; Whitelocke's "Sprains," 7s. 6d.; Brockbank's "Life Insurance and General Practice." All these latter volumes belong to the firm's "General Practice Series"—a collection which, although it varies in quality, is on the whole decidedly to be recommended. Among other volumes in this series we may mention Murphy's translation of Kirmisson's "Surgery of Children," and Hay's "Heart Disease—Graphic Methods," two interesting books which are well worth buying. Another excellent series is the Oxford Medical Manual Series, nearly every one of which is a concise summary of modern methods, written in a specially attractive manner, and, like all the works published by the firm, beautifully printed and illustrated. Among the new volumes and new editions in this series are Robson and Cammidge's "Gall-stones," Fowler's "Infant Feeding," Spencer's "Gunshot Wounds," Tod's "Diseases of the Ear," Mayous' "Diseases of the Eye," Lockwood's "Aseptic Surgery," Sargent's "Surgical Emergencies," and Barwell's "Diseases of the Larynx." Two volumes deserve special mention, as they ought to be on every practitioner's shelves; these are the admirable classic works of Dr. Gee, the one "Auscultation and Percussion," and the other "Medical Lectures and Clinical Aphorisms." In these modern days, when most medical authors write a jargon of their own, it is delightful to come across two such pure and unadulterated works as these. All the manuals are priced at 5s., and are very cheap at the price. The firm issues many other well-known works on medical, surgical, and scientific subjects, and now publishes such classics as Cunningham's "Anatomy," Crocker's "Atlas," and Mercier's "Criminal Responsibility." A full list of the books will be sent to any practitioner on application, and it is worth while noting that any book above 7s. 6d. in price will be sent on approval for examination.

#### Messrs. Churchill's New List.

J. and A. Churchill, of 7 Great Marlborough Street, is a well-known firm that publishes many old favourites. The new "System of Treatment," by Dr. Latham and Mr. Crisp English, which will be published early in 1911 in four large volumes, promises to be a standard work, and the list of contributors includes practically every name that is familiar in the consulting world. It is perhaps too big a series for the general practitioner, but merits attention at any rate. Leaving out the books manifestly written for students only, which, with the exception of such excellent works as Dr. Taylor's "Practice of Medicine," and Fleming's "Short Practice," both of which can be heartily commended as useful works, scarcely fall within the scope of this article, we may mention such really valuable productions of this firm as Spencer and Gask's "Practice of Surgery," one of the finest one-volume books on the subject we are acquainted with; Gallabin's "Midwifery," the new edition (written in collaboration with Dr. Blacker) of which has just been issued at 18s.; Eden's "Manual of Midwifery," and Jellett's "Short Practice," Firth's "Hygiene," 21s. net, and the new edition of Goodhart and Still's "Diseases of Children," one of the most popular works on the subject, which has been almost rewritten and brought thoroughly up to date.

Other interesting books published by this firm are Pollard's edition of Heath's "Minor Surgery," Parson's "Diseases of the Eye," Smith's new edition of that well-known classic Taylor's "Medical Jurisprudence," Clouston's "Mental Diseases," Craig's "Psychological Medicine," Hewlett's "Serum Therapy," and Dr. Sequiera's new "Handbook on Skin Diseases," which will be ready early in the new year. The firm issues an illustrated catalogue, which gives valuable information with regard to their books, and which is forwarded post free on application.

### Rebman's Books.

Another excellent firm is that of Rebman, Ltd., which makes a speciality of works more fitted for the practitioner than for the student, and of translations of standard foreign books. This firm publishes Dr. Judd's work on "Medical Electricity," and Belot's book on "Radiotherapy." It also issues many books on subjects of great interest to the thoughtful practitioner, and is well known, for instance, as the house that publishes the translation of Krafft-Ebing's work on "Psychiatry."

Messrs. Rebman's new and forthcoming medical publications include "Mental Symptoms of Brain Disease," by Dr. Bernard Hollander, just ready (reviewed in this number), 6s. net; "Practical Points in the Use of X-Ray and High-Frequency Currents," by Aspinwall Judd, M.D., 6s. net; "Hints for the General Practitioner in Rhinology and Laryngology," by Dr. Johann Fein, translated by Dr. Bowring Horgan, M.B., B.Ch., 5s. net; "The Diseases of the Nose, Mouth, Pharynx, and Larynx," by Alfred Bruck, M.D., 21s. net; "Diagnostic Therapeutics," by Abrams, 21s. net; "Sexual Life of Woman," by Kisch, 21s. net; "Anæmia," by Ehrlich Lazarus and Nageli, translated by H. W. Armit, 12s. 6d. net; and "Therapeutics of Ophthalmic Practice," by Dr. Curt Adam, of Berlin, translated by Dr. W. G. Sym, of Edinburgh; also the following: A large new work on the "Abdomen Proper," by Dr. W. Cuthbert Morton, of Leeds, consisting of twenty-eight anatomical plates, prepared on an entirely new, and it is believed novel, plan. Bing's "A Compendium of Regional Diagnosis in Diseases of the Brain and Spinal Cord"; "A Concise Introduction to the Principles Governing the Clinical Localisation of Diseases and Injuries of the Central Nervous System," by Robert Bing, Privatdozent for Neurology in the University of Basle, translated from the German by Francis Sorrell Arnold, M.B., B.Ch. Oxon., with seventy illustrations (in the press). "Disorders of Metabolism and Nutrition," by Professor Dr. Carl Von Noorden. Part VIII., "Inanition and Fattening Cures," 5s. net; Part IX., "Technique of Reduction Cures and Gout," 5s. net, both just issued.

### Wright and Sons.

John Wright and Sons, of Bristol, have lately published some very interesting practical works. In another column we review the second volume of Dickie's translation of Lejar's "Urgent Surgery," a work which should commend itself to all practitioners; it is thoroughly up to date and practical. The list forwarded by the firm includes such works as Dr. Fortescue Brickdale's "Practical Guide to the Newer Remedies," a work that should prove very useful in practice; Mr. Percival's "The Prescribing of Spectacles," and Dr. Maddox's "Golden Rules of Refraction"; Dr. Blair's "Errors in Refraction"; Clayton, Green and Cope's new edition of "Pye's Minor Surgery"—a well-known and deservedly popular little book; and many others. Messrs. Wright will shortly issue, in large octavo form, lavishly illustrated, Mr. Morrison's "Introduction to Surgery," which will be published at a moderate price. It is a work that should obtain a wide reading public, as it deals with treatment and diagnosis, and should appeal more directly to the practitioner. All the works emanating from the Bristol house are well printed and got-up, and form excellent and attractive additions to the library. Catalogues are forwarded free on application.

### Cassell and Co.

Elsewhere in this issue we publish a review of one of Messrs. Cassell and Co.'s latest issues—Wickham and Degrais' "Radiumtherapy," a book which is well worth a place on the general practitioner's library shelf. Many other good practitioners' manuals are printed by this firm. Among them we may instance the series of manuals under the general editorship of Sir Malcolm Morris, which is specially designed for practitioners. These include Bosanquet and Eyre's "Serum Therapy," Bramwell's "Hypnotism," Burton Fanning's "Open-air Treatment of Phthisis," Butlin and Spencer's "Diseases of the Tongue," Habershon's "Diseases of the Stomach," Goodall and Savage's "Insanity," Dr. Herman's useful manuals "Diseases of Women," and "Difficult Labour," Hutchinson's classical work on "Syphilis," Manson's "Tropical Diseases," Morris's "Diseases of the Skin" and "Light and X-ray Treatment," Owen's "Surgical Diseases of Children," Sutton's "Tumours," Thomson's "Diseases of the Nervous System," Treves' "Manual of Operative Surgery" (a magnificent new edition of which has lately been issued), and the same author's useful little manual of "Surgical Applied Anatomy" (a book that every practitioner should possess), Burney Yeo's "Manual of Medical Treatment" (a new and enlarged edition of which has been published also quite recently), and many other works. The volumes are all beautifully printed, and the illustrations everywhere clear and really helpful. A Cassel on the bookshelf is always an ornament, and it may be relied upon to be as useful as it is ornamental.

### Lewis' Library.

Mr. H. K. Lewis, 136 Gower Street, London, W.C., will publish early in November an entirely new work on "Medical Diagnosis," by Dr. Mitchell Stevens, of Cardiff. It will be illustrated by upwards of 175 illustrations, including several in colour, and will be in one volume. This firm also has in the press a new edition of Sir Douglas Powell's "Diseases of the Lungs"; in this, the fifth edition, Dr. Horton-Smith Hartley is collaborating; also a new book by Dr. Mildred Burgess on "The Care of Infants and Young Children in Health." Amongst recent publications may be mentioned a reprint of the second edition of Prof. William Osler's "Aequanimitas, with other Essays and Addresses to Students, Nurses, and Medical Practitioners."

Other recent issues include a second edition of Mr. Arthur Cooper's "Sexual Disabilities of Man and their Treatment"; the late Dr. Logan's posthumous papers in three volumes entitled "Vetera et Nova, Biological Physics, Physic and Metaphysic"; Dr. W. Gordon's monograph on "The Influence of Strong Prevalent Rain-bearing Winds on the Prevalence of Phthisis"; a reprint of Dr. Eustace Smith's popular essays "Some Common Remedies and their use in Practice"; the second volume, completing the fourth edition, of Binnie's "Operative Surgery"; a second edition of Dr. Percy Lewis's "Medical Exercises"; and last, but not least, the fourteenth edition of "The Extra Pharmacopœia" of Martindale and Westcott, with an important supplement by Dr. Harrison Martindale, "Organic Analysis Chart." This standard work was reviewed fully in our issue of September 17.

In Mr. Lewis's Bookselling Department a complete stock of recent medical and scientific literature is always open to inspection. The books are conveniently arranged under subjects. The reading-room in connection with the Circulating Library is also open daily to subscribers. All the more recent books are kept on the shelves in this room, and are thus readily accessible to subscribers.

### Longmans' List.

Longmans, Green and Co. are well known as medical publishers, and among the "practitioners'" books they list in their catalogues we may mention such old favourites as Ashby and Wright's "Diseases of Children," which remains perhaps the finest text-book on the subject in the language; Sir William Bennet's works, which are nearly all published by Longmans, are too familiar to practitioners to need an introduction here, while Cheyne and Burghard's "Manual of Surgical Treatment" is a standard authority—of which, by the way, we would like to see a second edition brought out. Dakin's "Handbook of Midwifery" is equally familiar, but a novelty which Messrs. Longmans are bringing out this autumn is Blair Bell's "Principles of Gynæcology," a handsome guinea volume, which is profusely illustrated, and which is an ideal work for the practitioner and the senior student. Another interesting novelty—though it can hardly be called a "practical handbook"—is "The Physiology of Reproduction," by Dr. Marshall, which is a comprehensive account of what we know regarding the physiology of the reproductive organs and the phenomena of generation and sex. Many other interesting works are given in this firm's list. Fowler and Godlee's "Diseases of the Lung" is there, our old friend "Gray," one of the best manuals of anatomy the practitioner can ever invest in; Quain's "Dictionary of Medicine," William's "Rhinology," and let us not forget to add Dr. Moon's philosophic "Relations of Medicine to Philosophy," Mr. Paget's "Memoir" of his father, and the large number of well-known scientific books that come from this firm. Messrs. Longmans' catalogue is a long and interesting one, and the practitioner building up his library cannot afford to disregard it.

### Macmillan's New Works.

Macmillans, though a "general" firm, which does not specialise in medical books to the extent that Longmans does, is nevertheless another important publishing house. Here in this firm's catalogue we come across that monumental work "Allbutt's System," which should be on the shelves of every practitioner who can afford to buy it, for it is a mine of information and a library almost in itself. The new edition, with the ample bibliographies at the end of each article, is thoroughly up to date, and really a splendid work. Less excellent, though very useful, is Allbutt, Playfair, and Eden's "System of Gynæcology," issued by the same firm. Among other Macmillan specialities we may mention Ballance's "Points in the Surgery of the Brain," Lauder Brunton's works, Copeman's "History of Vaccination," Hawkin's "Diseases of the Appendix," Stonham's "Surgery," Allchin's "Manual of Medicine," Mercier's "Nervous System and Mind," Richardson's classic work on "The Field of Disease," and Tubby and Jones' "Surgery of Paralysis." A glance through Macmillan's catalogue will show the practitioner that there are many other works, not immediately to be classed under medicine and surgery, that ought to find a place on his shelves.

### Messrs. P. S. King and Son.

Messrs. P. S. King and Son have lately published several good works, mostly belonging to the domain of preventive and State medicine. Dr. Kelynack's book on "Medical Examination of Schools and Scholars," lately reviewed in these pages, comes from this house. Sir Isaac Pitman and Sons have also lately issued several interesting books, among which we may instance Dr. Bernard Hollander's "Hypnotism and Suggestion," reviewed in The Hospital last month. Messrs. Constable and Co. announce two new works, both by Mr. William Battle. The one is on the "Acute Abdomen," and the other deals with the "Surgery of the Appendix." Both appear to be very suitable for practitioners.

### Various Other Firms.

A word must be said about the useful anatomical and other models and charts which are published by Messrs. George Philip and Son, of Fleet Street. These are well worth some attention on the part of the practitioner. A country doctor may often be called upon to give first-aid lectures, especially in these days of voluntary detach-

First Aid to the Injured. Plate 2.

ments, and it is always well to know where one may procure the best works for demonstration purposes. Philip's catalogue will therefore prove a great help. We herewith give a much reduced illustration, showing one of his first-aid diagrams, which, printed in colours and mounted on rollers, map style, are published at the low price of 4s. each. An excellent work, which can be cordially recommended to practitioners is Dr. Cautley's

"Manual of Diseases of Infants," published by Shaw and Sons, at the price of one guinea. It is quite a new work, having been issued this year, and ranks as one of the finest English works on the subject.

Messrs. Nisbet and Co. and Messrs. Baillière, Tindall and Cox are very well known medical publishers. The former's list includes a new edition of Bland Sutton's "Gall Stones," Wallis's "Surgery of the Intestines," Berry's "Hip Disease," and Milne's "A Plea for the Home Treatment of Scarlet Fever." Nisbet's "Medical Directory," which, in our opinion, is the ideal directory for the profession, is issued by this firm. Messrs. Baillière, Tindall and Cox announce the following autumn works: "Manual of Physiology," by G. N. Stewart, 6th edition, 18s. net; "Military Hygiene," by Lieut.-Col. R. Caldwell, 2nd edition, 12s. 6d. net; "After-Results of Abdominal Operations," by A. E. Giles, 7s. 6d. net; "Intestinal Surgery," by L. A. Bidwell, 2nd edition, 6s. net; "Gynæcological Therapeutics," by S. J. Aarons, 5s. net; "Veterinary Parasitology," by R. H. Smythe, 5s. net; "Physiological Principles in Treatment," by W. L. Brown, 2nd edition, 5s. net; "Syphilis: Its Diagnosis and Treatment," by Col. F. J. Lambkin, 5s. net; "Some Considerations of Medical Education," by S. Squire Sprigge, 2s. 6d. net; Knocker Douglas, "Accidents in their Medico-Legal Aspect," 25s. net; E. M. Steven, "Medical Supervision in Schools," 5s. net. This firm also publishes Dr. Walsh's book on the hair, which we review on the next page.

Homœopathic practitioners will find most interesting material for selection in the catalogues of the Homœopathic Publishing Company, which is issuing new editions of the well-known works of Clarke Ruddock and Burnett.

---

Urgent Surgery. By Felix Lejars. Translated from the sixth French edition by William S. Dickie, F.R.C.S. Volume II. (Bristol: John Wright and Sons. London: Simpkin, Marshall, Hamilton, Kent and Co., Ltd. Price 25s. net.)

Some months ago we had pleasure in reviewing Mr. Dickie's admirable translation of the first volume of this monumental work, and we then expressed the hope that the forthcoming second volume would be as good as the first. With the second volume before us we are in a position to say that that hope has been amply realised. These two volumes, representing not only a fair and full translation of Lejars' well-known work, but Mr. Dickie's notes and excellent editing, are among the finest surgical works produced in this country during the last ten years. We say so deliberately, with a knowledge of nearly all the works on surgery that have been published during that period. This volume is fully equal to the first in point of interest and excellence of translation. Mr. Dickie has done his work ably, and he reads, as in the first volume, everywhere fluently. There are no jarring sentences and no literals that abominate by their literalness, though here and there one meets with words that are not fully naturalised. Cerclage of the patella is one of these; we see no reason for discarding the expressive "ringing of the patella," whether with wire or tendon, that old surgeons still frequently use. But these are minor matters, and our concern is mainly with essentials. The volume deals with the genito-urinary organs, the rectum and anus, strangulated hernia, and with the extremities. Every section is abundantly and practically illustrated, and those who know Lejars only in the 1906 and earlier editions will find many additions to the text and a mass of valuable material enshrined in the footnotes. A feature of the work is, of course, the full descriptions of the operations, in each case accompanied by a short summary of the indications for operation. In many books the student is perplexed, and the practitioner who is face to face with an emergency often irritated, by the vacillating manner in which these works, meant to serve as guides for them, discuss these indications for operation. While free and open debate is admirable enough in articles intended for specialists or those who are in a position to compare and criticise, a certain amount of dogmatism—or let us rather say a certain amount of clinical cocksureness, such as a student expects from a master who has had a large amount of experience—is desirable and necessary. Lejars has adopted this method to a large extent, and although he leaves his readers a large amount of choice, there is never any doubt as to which side of a question his own opinion inclines. This is well seen in the chapter dealing with renal decapsulation and nephrotomy. Here and there the practice he recommends will not appeal to English surgeons. He favours the cautery knife at a red heat for incisions into the scrotum and perineum in cases of extravasation, and advises against drainage in this condition. While this will be novel treatment to some of us, he does not hesitate to defend it, and his arguments are well worth study. No form of drainage is of any value, and it is best to leave the incisions widely open and cover the area of extravasation with a layer of moist gauze. This is certainly in agreement with the experience of many surgeons, although it is not the routine English practice. The thermo-cautery, Lejars remarks, is to be preferred to an ordinary scalpel, because it not only prevents loss of blood but also exercises a special and salutary action on the infiltrated and devitalised tissues. This seems to us rather far fetched. The scalpel makes a cleaner wound, and bleeding is, after all, not greatly to be feared in these cases, while the pain and after-shock of the cautery knife probably largely counteract any supposed salutary effect it may have. The sections dealing with urinary troubles and operations generally are clear and well written, with plenty of illustration in the form of typical cases: so, too, are the chapters on the rectum and anus. The notes on urgent anal dilatation in cases of strangulated piles are well worth reading, as the operation is one that can easily be carried out by the general practitioner, and one also that yields immense relief to the patient. Operations for hæmorrhoids, not falling under the category of urgent surgery, are not discussed. The most interesting chapter, and one of the most valuable, is undoubtedly that on strangulated hernia. Obviously par excellence the operation of urgency which the practitioner can and ought to master, the simple details of the various methods are often given in an involved and uninteresting way in ordinary text-books. While no one should attempt the operation who has not had an opportunity of at least seeing it done, Lejar's notes will be of the greatest service to those who, although they have seen a strangulated hernia operated on, and may even have assisted at the operation themselves, have but dim impressions of the method used, and are suddenly called upon to relieve a patient. It is for such practitioners that this book is specially written, and obviously the fairest test to apply to it is its practical value in everyday emergencies. After carefully reading the section on strangulated hernia we can safely say that it is one of the finest and most directly practical chapters we have come across, reminding one at times of the classical work of Reclus on the subject. The instructions given are to the point, there is no wavering between two or more lines of attack, the reader is told what to do, and how to do it in the clearest, simplest, and most practical way. The illustrations greatly help the translator and author in their treatment of the subject. The remaining chapter deals with urgent operations on the extremities—dislocations and fractures, arthrotomies of urgency, burns and scalds, injuries to the soft parts and blood vessels, abscesses and so on. It is necessarily a widely diversified chapter, dealing as it does with many different lesions and their treatment, and the methods advocated will not all commend themselves to those who have had no experience of the French school. The notes on diffuse cellulitis are particularly good, and hardly open to this objection. The author lays great and deserved stress on the importance of early and heroic incisions: here, as in the case of urinary extravasation, he recommends the use of the thermo-cautery, and advises the simple warm bath for immersion of the limb without the addition of any antiseptics. Afterwards he uses peroxide of hydrogen or alcohol dressings. In cases of anthrax he advises excision of the pustule by the thermo-cautery, with subsequent injections of tincture of iodine around the site. This method is excellent, and certainly gives as good results as any other, while it has the advantage of being easily available and necessitating no expensive sera. The notes on fractures and dislocations are useful, and the photographs illustrating them of particular value, since some of the apparatus used will be unfamiliar to English readers. The section on open reductions is equally full and practical. Although it is impossible for us to review this work with the fulness that its practical utility and excellence merit, we have said sufficient to show that it is a work-book that is of the greatest value to the general practitioner. It is essentially a volume for reference and use in cases of urgency and emergency, just those occasions where the ordinary text-book so often fails by reason of its uncertainty or want of decisiveness. This work will, therefore, be found to supply a real want in a thoroughly up-to-date fashion.

The Mental Symptoms of Brain Disease. An Aid to the Surgical Treatment of Insanity due to Injury, Hæmorrhage, Tumours, and other Circumscribed Lesions of the Brain. By Bernard Hollander, M.D., with preface by Dr. Jul. Morel, late Belgian State Commissioner in Lunacy. (London: Rebman, Limited. 1910. Pp. 237. Price 6s. net.)

In his efforts to provide an introduction to the study of the initial stages of insanity the author has produced a book which should prove of the greatest interest to all classes of practitioners. It has been his aim to set forth the results of focal lesions only, for he leaves entirely on one side insanity due to general affections, brain degeneration, or toxins. In other words, he deals solely with those forms of insanity which are curable by removal of the cause of the mental disturbance. He points out the errors into which physiologists may fall in attempting to explain psychic phenomena in the light of their experience from experiments on the lower animals, and he emphasises the importance of the conclusions which may be arrived at from the study of clinical cases and post-mortem findings. His book is a collection of records of cases reported by numerous observers, himself among this number. While each case is reported in sufficient detail to entitle us to draw conclusions from it, references are given in all reports enabling the student to obtain fuller details if he wishes. Each portion of the brain is dealt with in turn, and the mental symptoms arising from lesions of the respective portions are set forth in detail. Such symptoms as arise in man from these lesions are then contrasted with those occurring in lower animals from analogous injuries artificially produced. The book should meet with a hearty reception, for it fills a gap in that portion of medical literature with which it is connected.

The Hair and its Diseases. By David Walsh, M.D. (London: Baillière, Tindall and Cox.

Although the hairy scalp is quite obviously but a specialised portion of the general cutis of the body, the treatment of its abnormalities has fallen to a great extent into the hands of ignorant quacks and self-styled beauty specialists. This is in part the fault of the medical profession, which has taken too little interest in the scientific study of the hair and its diseases, and in part the result of the feverish anxiety of mankind—especially the female sex—to retain or regain those physical attractions of which a head of beautiful hair forms so important a component. As long as knaves are allowed to exploit this perpetual human characteristic for their own gain, so long will the reproach attach to the medical profession of neglecting a branch of medicine which is as important to the mental well-being of the community as the study of, say, heart disease is physically. Of the book now under review it may be said that, although elementary in design, it conveys much that the general practitioner is ignorant of or ignores, to the detriment of his own credit and his patient's comfort. Especially important are the sections dealing with the hygiene of the barber's shop. The suggestions laid down as to the proper care of razors, hair-brushes, and similar toilet accessories might well be applied also in the home. Unfortunately, as far as the barbers' shops are concerned, to follow out antiseptic precautions on a rational scale must necessarily involve increased charges. Now, the public resents this, and is not yet sufficiently educated to prefer the hygienic but more expensive barber to his less scrupulous and cheaper opponents. Hence the barber who takes his art seriously is likely to find himself deprived of that measure of public support without which he cannot continue to make a living. The remedy is, of course, the instruction of the public in hygiene; and this is a long, tedious, and difficult task. It is a task, however, which the conscientious medical man can do a great deal to lighten, if only he will act as a health-missioner on every reasonable occasion. As a means of supplying him with information which he ought to possess, this small book fills a distinct gap in medical literature. The second edition has been brought well up to date by the inclusion of electrical and similar measures of physical therapeutics, and can be recommended as an inexpensive and trustworthy guide to the subject with which it deals.

A Manual of Chemistry, Adapted to the Requirements of Students of Medicine. By Arthur P. Luff, M.D., B.Sc.Lond., F.R.C.P., F.I.C., and Hugh C. H. Candy, B.A., B.Sc.Lond., F.I.C. (London: Cassell and Co., Ltd. 1910. Pp. 622, with 46 illustrations. Price 7s. 6d. net.)

No one would pretend that a comprehensive grasp of chemistry could be obtained from the study of only such portions of that science as have a practical bearing on medicine. This does not mean to say, however, that it is inadvisable to teach chemistry from a clinical point of view, and it is precisely this task which the authors of the present volume have set themselves. Catering especially, but not exclusively for the medical student, it has been their object to prepare as complete an introduction to the general theory and practice of chemistry, as could usually be compassed by a student of the science in his first year; but on the other hand, examples of clinical interest or importance are more frequent than they would be in a similar work intended for the general student. The appearance of a new edition of the book has been utilised largely to rewrite and bring it up to date, and from what we have seen of it we feel sure that it will be found an exceedingly useful introduction to chemistry.

## Figures and Tables

**Figure f1:**